# Sagittal biplanar ‘L’-shaped tibial osteotomy associated with osteochondral allograft for symptomatic varus knee and low patella

**DOI:** 10.1016/j.ijscr.2025.112074

**Published:** 2025-10-21

**Authors:** Eliseo Javier Firman, Eduardo Manuel Río, Adrián Nicolás Sirio

**Affiliations:** a Instituto Argentino de Diagnóstico y Tratamiento (IADT), Buenos Aires, Argentina; b Grupo Médico Teuos, Buenos Aires, Argentina

**Keywords:** “L”-shaped tibial osteotomy, Genu varum, Low patella, Osteochondral transplantation, Allograft, Patellar height, Joint preservation surgery

## Abstract

**Introduction:**

The “L”-shaped tibial osteotomy is a relatively recent surgical technique aimed at correcting the mechanical axis without altering patellar height or posterior tibial slope (common complications associated with conventional medial opening wedge osteotomies).

**Case presentation:**

We report the case of a young patient with symptomatic genu varum, osteochondral lesion in the medial femoral condyle, and low patellar height. A sagittal biplanar “L”-shaped proximal tibial osteotomy was performed, fixed with a Puddu plate and two anteroposterior screws. The osteotomy gap was filled with non-irradiated morselized bone allograft to promote consolidation. Additionally, a fresh osteochondral allograft transplant was carried out in the same surgical procedure.

**Clinical discussion:**

This technique allows for multiplanar correction while avoiding the increase in posterior tibial slope or reduction in patellar height, commonly seen with standard approaches. In our experience, combining dual anteroposterior screw fixation, morselized allograft, and osteochondral transplantation in a single stage resulted in improved mechanical stability, early bone integration, and satisfactory functional recovery.

**Conclusion:**

The biplanar “L”-shaped tibial osteotomy offers a theoretical biomechanical advantage by enabling coronal correction without altering the posterior tibial slope or patellar height in the sagittal plane. In this case, it allowed simultaneous management of the deformity and the osteochondral lesion in a single-stage procedure. However, as this is a single case, the results should be interpreted with caution and require confirmation through larger series with long-term follow-up.

## Highlights

• Novel use of sagittal L-shaped tibial osteotomy with fresh allograft
• Preserves patellar height and posterior tibial slope in varus knee correction
• Combined in one-stage with osteochondral allograft for focal cartilage lesion
• Early bone healing and functional recovery achieved at 12-month follow-up
• New Technique avoids common complications of standard medial opening HTO.

## 1 Introduction

Symptomatic genu varum in young adults is uncommon but can lead to pain, malalignment, stiffness, and significant functional limitation. Proximal tibial valgus osteotomy is an effective treatment option, as it allows realignment of the mechanical axis of the lower limb and redistributes weight-bearing load from the medial compartment toward the central zone [1,2].

Among the available techniques, medial opening-wedge osteotomy is widely used due to its precision in angular correction and the fact that it avoids additional intervention on the fibula, thereby reducing the risk of common peroneal nerve injury. However, it also presents some biomechanical drawbacks: it may cause patellar descent (patella baja), leading to anterior chondral wear and technically complicating any future total knee arthroplasty [1]. Additionally, this technique often increases the posterior tibial slope, which places additional strain on the anterior cruciate ligament (ACL) and may compromise its function [2].

To avoid patellar descent, a distal osteotomy below the tibial tuberosity (TT) has been proposed. However, this variant involves predominantly cortical bone cuts, which complicate fixation and carry a potential risk of delayed bone healing [1].

In this context, we present a surgical alternative for the correction of symptomatic genu varum in young patients: a proximal tibial sagittal biplanar “L” osteotomy that includes the tibial tuberosity in the proximal fragment. This technique allows for coronal realignment without altering patellar height or posterior tibial slope in the sagittal plane. Although the ‘L’-shaped osteotomy has been previously described in the literature, there are no reports documenting its combined application with osteochondral allograft transplantation in a single-stage procedure, together with the use of morselized bone allograft at the osteotomy site and fixation with two anteroposterior screws, as performed in this case.

## 2 Case presentation

We present the case of a 36-year-old male patient with progressive pain in the medial compartment of the left knee, associated with functional limitation and clinically evident varus malalignment. As a surgical history, the patient had undergone a medial partial meniscectomy five years earlier at another institution.

On physical examination, he presented localized pain in the medial compartment, mild stiffness, and inflammation without significant joint effusion. No signs of ligamentous instability were found. The patient had no relevant comorbidities and no history of regular medication use.

**Fig. 1:**
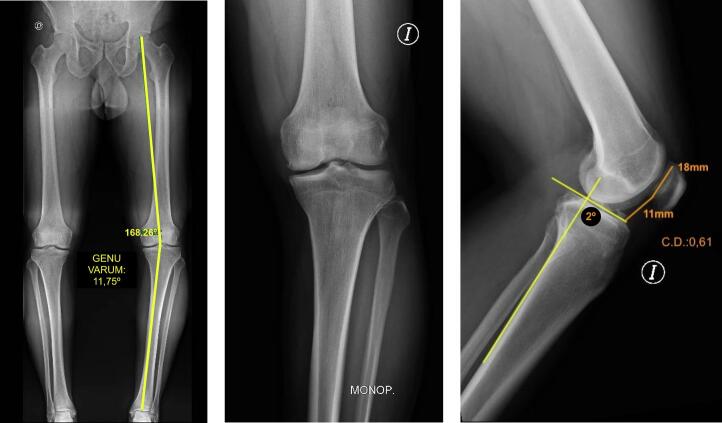
Preoperative measurements: A) Long-leg alignment radiograph showing genu varum. B) Anteroposterior monopodal weight-bearing radiograph. C) Measurement of posterior tibial slope and Caton-Deschamps index.

Imaging studies revealed early signs of medial femorotibial osteoarthritis, with an osteochondral lesion in the medial femoral condyle. Full-length weight-bearing radiographs (standing alignment radiographs) showed a mechanical axis deviation consistent with 11.75° of genu varum in the left knee (Fig. 1).

A lateral knee radiograph in 30° of flexion was used to assess patellar height, which measured 0.61 according to the Caton-Deschamps index [3]. This index was selected because it is independent of knee flexion angle, skeletal maturity, or postoperative patellar bone remodeling. Based on previous reports, the indication for a sagittal “L”-shaped osteotomy is considered when the Caton-Deschamps index is ≤1, depending on the degree of wedge opening required [2]. In the same projection, posterior tibial slope was measured as the angle between a line along the posterior tibial cortex and a line parallel to the tibial plateau [4]; the measured slope was 2 degrees (Fig. 1).

A Merchant axial view radiograph, obtained with the knee flexed at 45°, was used to calculate the lateral patellar tilt (LPT) angle and to assess the severity of patellofemoral osteoarthritis according to Merchant's criteria [5]. The measured preoperative LPT angle was 3.48°, without radiographic signs of advanced patellofemoral degeneration.

The Rosenberg view showed a 2 mm joint space narrowing in the medial femorotibial compartment, consistent with significant degenerative changes.

Computed tomography revealed cortical irregularities in all three compartments of the knee, with marginal osteophyte formation and reduced medial femorotibial joint space. Magnetic resonance imaging confirmed chondral degeneration in both the medial tibiofemoral and patellofemoral compartments, along with marginal osteophytosis, joint space narrowing, and reduced volume of the medial meniscus.

A proximal sagittal “L”-shaped valgus tibial osteotomy was planned, combined with non-irradiated morselized bone allograft at the osteotomy site and an osteochondral allograft transplant for the medial femoral condyle lesion. The surgical goal was to correct the femorotibial axis as close as possible to the anatomical alignment (5–7° of valgus), shifting the mechanical load to the Fujisawa point [6], while simultaneously preserving patellar height and posterior tibial slope.

## 3 Surgical technique

The patient was placed in the supine position under regional anesthesia (ultrasound-guided posterior popliteal sciatic block) combined with general anesthesia. A pneumatic tourniquet was applied with a pressure of 300 mmHg.

The procedure began with diagnostic arthroscopy to evaluate all three knee compartments, stage the cartilage lesions, and perform debridement, synovectomy, and partial resection of degenerated residual meniscal tissue. Chondral lesions were classified according to the International Cartilage Repair Society (ICRS) grading system [7].

A longitudinal skin incision of approximately 5 to 7 cm was made on the proximal anteromedial aspect of the leg, starting 1 cm below the joint line and extending distally between the tibial tuberosity (TT) and the posteromedial border of the tibia. Layered dissection was carried out, the hamstring tendons (semitendinosus and gracilis) were released, the distal insertion of the superficial medial collateral ligament was detached, and the tibial periosteum was elevated. The patellar tendon was protected using a retractor, and the proximal 3 cm of the tibial tuberosity were exposed.

Under fluoroscopic guidance, a 2 mm Kirschner wire was inserted from the medial tibial cortex to the lateral side at the metaphyseal–diaphyseal junction, in an ascending oblique direction of approximately 30°, terminating 1 cm proximal to the tibiofibular notch, preserving the native anatomical corridor between the tibia and fibula.

**Fig. 2:**
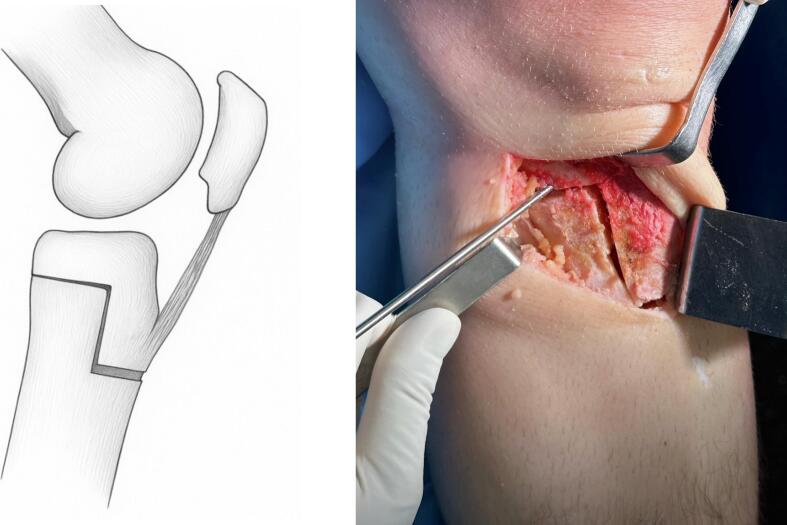
Lateral view of the sagittal “L”-shaped osteotomy.

An “L” shape was marked on the medial cortex of the proximal tibia using electrocautery. Guided by the wire and cortical markings, the osteotomy cuts were performed using an oscillating saw, osteotomes, and chisels (Fig. 2).

The horizontal cut is similar to that used in standard medial opening-wedge valgus tibial osteotomy, whereas the vertical cut must be made posterior to the tibial tuberosity.

The first cut, in the coronal plane, was made from medial to lateral at approximately 30° of obliquity, leaving the outermost lateral centimeter of cortical bone intact to serve as a hinge. In the sagittal plane, this cut was made perpendicular to the longitudinal axis of the tibia to preserve the posterior tibial slope (PTS).

The second, vertical cut was made posterior to the TT, perpendicular to the first cut, extending 3 to 5 cm distally, isolating the tibial tuberosity from the tibial shaft but keeping it attached to the proximal fragment. The TT thickness was maintained at a minimum of 1 cm to reduce fracture risk and allow for screw fixation without difficulty. Finally, a third horizontal distal-anterior cut was made to complete the osteotomy. This preserved the tibial tuberosity as part of the proximal osteotomized segment.

Valgus force was applied to open the osteotomy wedge according to the preoperatively calculated correction angle. A posterior wedge was placed at the dorsal cortical surface of the osteotomy to maintain a trapezoidal gap with greater posterior height, which helps prevent an increase in the posterior tibial slope.

Before positioning the osteosynthesis plate, two 3.5 mm cortical screws were inserted in an anteroposterior direction through the tibial tuberosity (TT) to prevent anterior displacement during wedge opening, which could otherwise increase the posterior tibial slope. The use of two screws is recommended to avoid stress shielding phenomena that may lead to implant deformation or failure.

Osteosynthesis was performed using a Puddu plate (a proximal tibial anatomical plate may also be used) and 4.5 mm locking screws. A 3.5 mm cortical screw was initially placed to approximate the plate to the bone, which was later replaced after the locking screws were inserted. After wedge removal, the osteotomy gap was filled with ground cortico-cancellous, fresh-frozen, non-irradiated allograft prepared with 1 g of vancomycin per 50 cc, provided by the tissue bank, stored at −80 °C, and thawed under sterile conditions immediately before implantation.

The hamstring tendons and superficial medial collateral ligament were repositioned and sutured to cover the plate. The surgical wound was closed in anatomical layers.

Subsequently, a medial arthrotomy with an anterior approach was then performed to expose and measure the osteochondral lesion of the medial femoral condyle, allowing for planning of graft size and number. Reconstruction was carried out using a fresh-frozen, non-irradiated osteochondral allograft obtained from a cadaveric femoral condyle, shaped to fit the defect and implanted using a press-fit technique.

**Fig. 3:**
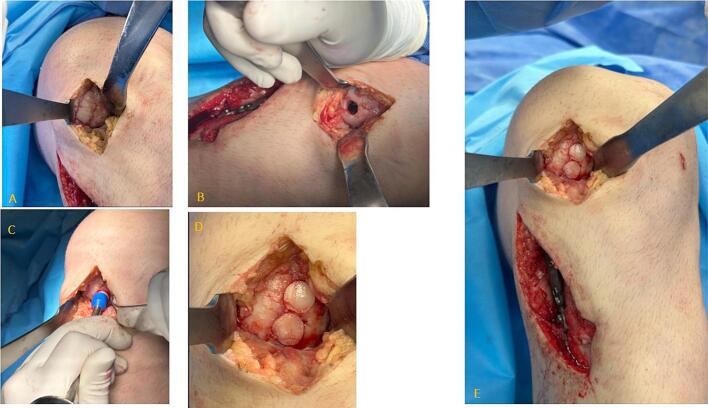
Osteochondral allograft transplantation in the medial femoral condyle: A) Initial chondral lesion. B) Preparation of the recipient bed. C) Impaction of cylindrical grafts. D and E) Final result of the osteochondral allograft transplantation.

The transplantation was carried out using small-diameter osteochondral plugs to better match the curvature of the articular surface. The plugs were placed with minimal spacing (approximately 1 mm) between them, at depths ranging from 5 to 15 mm, and impacted until flush with the native articular surface. Finally, the arthrotomy wound and arthroscopic portals were closed in layers (Fig. 3).

### 3.1 Rehabilitation protocol

Within 24 h after surgery, the patient began active ankle mobilization exercises, isometric quadriceps exercises as tolerated, and continuous passive motion (CPM) for knee flexion-extension.

During the first postoperative week, partial weight-bearing was allowed using forefoot support and maintained until week 6. From week 6 to week 8, the patient was gradually advanced to full weight-bearing.

### 3.2 Postoperative complications

As a postoperative complication, one month after surgery the patient required two surgical debridements due to a superficial wound infection, which was successfully treated with a six-week course of antibiotic therapy. This event did not negatively affect the clinical course, did not delay bone consolidation, and did not compromise the final functional outcome, as it was identified and managed early, including the aforementioned debridements.

No other complications were observed, such as deep vein thrombosis, delayed bone healing, or wound-healing disorders.  

**Fig. 4:**
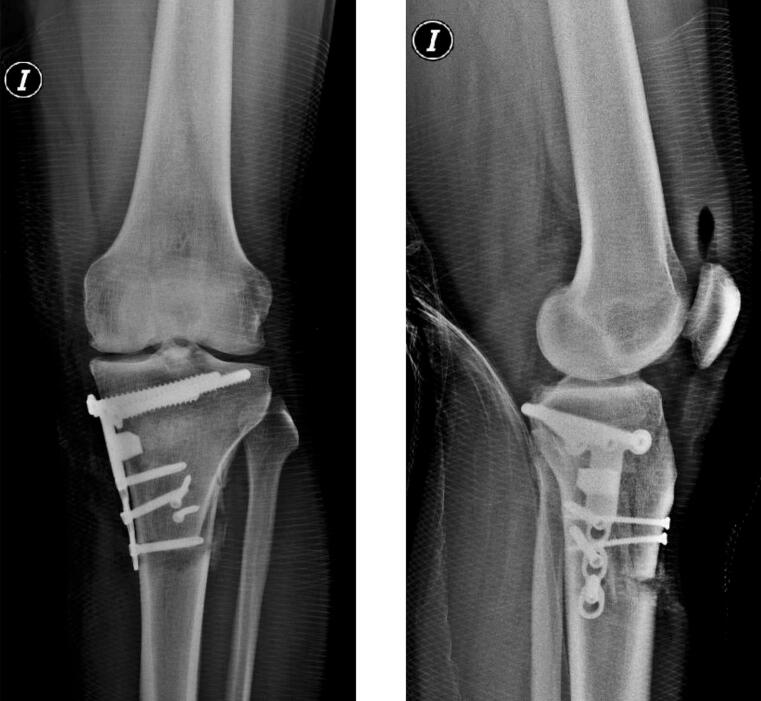
Postoperative radiographic follow-up of the “L”-shaped tibial osteotomy.

Imaging follow-ups were performed at 1, 3, 6, 8, and 12 months postoperatively. Osteotomy consolidation was confirmed at 12 weeks through plain radiographs and computed tomography (Fig. 4).

During the initial diagnostic arthroscopy, a grade 3 chondral lesion was identified in the patella and trochlea, and a grade 4 lesion in the medial femoral condyle. All lesions were classified intraoperatively according to the International Cartilage Repair Society (ICRS) grading system [7].

### 3.3 Reintervention and six-month evaluation

At six months after the initial procedure, the patient underwent a second surgical intervention for removal of the osteosynthesis hardware and joint debridement, including intra-articular fluid and tissue sampling for culture. Microbiological results were negative, ruling out active infection.

During the exploratory arthroscopy (second-look), the previously diagnosed chondral lesions in the patella and trochlea showed no significant progression and remained stable according to the International Cartilage Repair Society (ICRS) grading system [7]. The osteochondral allograft implanted in the medial femoral condyle showed proper integration, with no signs of detachment or degeneration.

**Fig. 5:**
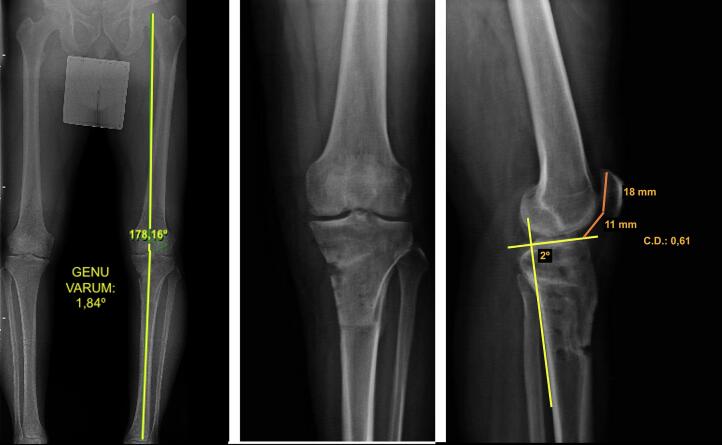
Final postoperative measurements: A) Long-leg alignment radiograph showing correction of varus deformity. B) Monopodal anteroposterior weight-bearing radiograph. C) Measurement of posterior tibial slope and Caton-Deschamps index.

Following hardware removal, patellar height was reassessed radiographically using the Caton-Deschamps index, which remained unchanged from the preoperative value (0.61). Similarly, the posterior tibial slope was measured at 2°, consistent with the preoperative measurement (Fig. 5).

Postoperative Merchant view radiographs showed a lateral patellar tilt (LPT) angle of 3.44°, indicating that the osteotomy did not produce changes in posterior tibial slope, patellar height, or lateral patellar tilt. These parameters remained stable at both 6 and 12 months of follow-up.

In the postoperative long-leg radiograph (telemetry) evaluation, an adequate correction of the mechanical axis of the lower limb was observed, changing from 11.75° of varus preoperatively to a residual varus of 1.84° at 6 weeks after surgery (Fig. 5).

### 3.4 Functional evaluation

At final follow-up, 12 months after surgery, the patient scored 86 on the Lysholm Knee Scoring Scale and 91 on the Kujala Anterior Knee Pain Scale, indicating satisfactory functional recovery.

Preoperatively, the patient reported moderate patellofemoral pain. At final follow-up, no anterior knee pain was reported.

## 4 Discussion

The original “L”-shaped tibial osteotomy technique was first described by Dr. Monllau and colleagues in 2017 [2], followed by a published case series of 23 patients in 2019 [8]. Despite its potential, the literature on this technique remains very limited.

In our experience, we have introduced relevant modifications that optimize the outcomes. First, we incorporated morselized cortico-cancellous allograft, fresh-frozen and non-irradiated, into the osteotomy site with the aim of promoting early consolidation, and complemented it with a fresh-frozen osteochondral allograft to address the chondral lesion of the medial femoral condyle.

The evidence regarding the use of morselized allograft in the osteotomy wedge is heterogeneous; however, several studies have shown that filling the gap with graft accelerates radiographic consolidation and return to weight-bearing. In a recent cohort [9], patients who received allograft achieved consolidation at an average of 21 weeks compared to 32 weeks without graft, and reached full weight-bearing at 8.2 weeks compared to 9.8 weeks.

Garabano et al. [10], although not studying osteotomies, demonstrated in tibial plateau fractures that non-irradiated allograft achieved a 97.4 % incorporation rate with minimal loss of reduction, reinforcing its validity as a metaphyseal filler. Autograft remains the biological gold standard but entails donor-site morbidity and limited availability; in contrast, allograft preserves osteoinductive and osteoconductive properties, as also reported by the same authors [10].

Mao et al. [11] observed higher consolidation rates with autograft in osteotomies with wide openings (mean 12.1 mm); however, they used lyophilized and sterilized allografts, whose processing reduces biological and biomechanical capacity, which likely explains their lower integration.

In our case, the fresh-frozen allograft, non-irradiated and non-lyophilized, avoided donor-site morbidity and better preserved tissue properties, favoring a more predictable integration. We employed morselized cancellous graft as an osteoconductive scaffold to reduce micromotion and promote early consolidation, in accordance with reports showing shorter union times and outcomes comparable to autograft [12].

We also adapted the fixation to our Latin American regional context: while the original series [2,8], systematically employed a single anteroposterior screw, in our case we opted to use two screws. The original description [2], considered this possibility, although it had not previously been reported in clinical practice. Our decision was based on the heterogeneity in the mechanical strength of locally available implants, where a single screw may not provide sufficient stability, exposing the patient to risks of secondary deformation due to mechanical stress. Double fixation represents a rational and complementary strategy to that proposed by Monllau [2,8], optimizing fragment stability and reducing the risk of micromotion.

Although no published biomechanical studies directly compare fixation with one versus two anteroposterior screws in the ‘L’-shaped tibial osteotomy technique described by Monllau et al. [2], data from analogous osteotomy and fixation studies support the concept that the use of multiple screws or supplemental fixation improves stability. For example, Yang et al. [13], demonstrated in an in vitro biomechanical study that adding a supplemental screw in medial open-wedge high tibial osteotomy (HTO) provided greater mechanical strength, less loss of reduction, and improved durability under dynamic cyclic loading compared with the standard treatment without the additional screw.

In tibial tubercle osteotomies, Chang et al. [14], demonstrated through a numerical model that the most stable configuration is two parallel horizontal screws, as this reduces fragment displacement and gap opening. If this option is not feasible in a clinical case, the downward configuration is recommended. Conversely, the two parallel upward screw configuration is not advisable for TTO fixation. Therefore, the use of two screws in our adaptation not only represents a logical response to implant availability but also aligns with biomechanical principles that favor greater rotational stability and reduced micromotion.

The use of two anteroposterior screws together with the incorporation of non-irradiated morselized allograft allowed for earlier bone integration, which facilitated initial functional loading and the anticipated yet timely removal of the osteosynthesis material.

From a biomechanical standpoint, the “L”-shaped osteotomy enables precise multiplanar correction with broad control of the mechanical axis, without compromising the posterior tibial slope or causing patellar descent. In our case, the technique did not significantly alter patellar height or patellofemoral joint congruence. The realignment of the mechanical axis was effective, with favorable clinical outcomes and an early recovery, largely attributable to the prompt consolidation of the bone graft.

Moreover, combining this tibial osteotomy with osteochondral allograft transplantation in the medial femoral condyle allows for simultaneous correction of both axial deformity and focal chondral injury, which offers a significant advantage over sequential approaches.

It is well documented that classic supratuberosity valgus tibial osteotomies, especially those involving greater angular correction, may lead to significant patellar height reduction—up to 20 %—increasing contact pressure on the inferior patella and due to patellar tilt. Clinically, this can manifest as anterior knee pain, flexion-extension limitations, and accelerated progression toward patellofemoral osteoarthritis [1,2,8,15,16]. The greater the correction angle, the higher the risk of increased posterior tibial slope and decreased patellar height [16].

In this regard, Erquicia et al. [8], highlighted that the biplanar “L”-shaped osteotomy avoids distalization and lateralization of the tibial tuberosity, enabling accurate correction of patellar axial malalignment. Therefore, it is also indicated in patients with symptomatic genu varum associated with radiologic signs of patellofemoral joint dysfunction, as it helps reduce patellofemoral overload.

Conversely, infratuberosity uniplanar osteotomies involve a large cortical bone surface, which can hinder fixation and reduce resistance to rotational forces, increasing the risk of delayed bone healing [1,2]. In contrast, the biplanar “L”-shaped osteotomy engages a broader cancellous bone area and provides a more favorable anatomic configuration for stable fixation with improved rotational control.

The standard supratuberosity valgus osteotomy technique is contraindicated in patients with low patella or patella infera. According to the Caton-Deschamps index, patella baja is defined as values between 0.6 and 0.8, and patella infera as values below 0.6. However, there is no clear consensus on the exact threshold at which the supratuberosity approach should be contraindicated, despite strong evidence that the procedure tends to lower patellar height [8].

In our practice, we selected the sagittal biplanar “L”-shaped valgus osteotomy technique in patients expected to have a postoperative Caton-Deschamps index below 0.8, aiming to minimize the risk of patella baja—an often unpredictable complication of conventional medial opening wedge techniques.

It is also well known that posterior tibial slope (PTS) tends to increase after medial opening wedge osteotomy and decrease after lateral closing wedge osteotomy. Increased PTS elevates anterior tibiofemoral contact pressure and reduces posterior femoral load, accelerating joint degeneration while also increasing strain on the cruciate ligaments, thereby affecting overall knee stability [2,8,16,17].

MacLeod et al. [17] identified two key factors influencing PTS changes during high tibial osteotomy: the opening wedge angle and the hinge axis orientation. They found that for every 10° of hinge axis inclination, there is approximately a 1° change in posterior tibial slope. They also demonstrated that posterior slope can be reduced even in medial opening wedge procedures, depending on hinge orientation.

Similarly, Chung et al. [18] evaluated the effect of sagittal osteotomy inclination and found that anteriorly inclined osteotomies tend to increase posterior slope, while posteriorly inclined osteotomies reduce it. The change in PTS was proportional to the angle of sagittal cut.

Based on these findings, Monllau et al. [2] recommended in 2017 that the anterior opening height of the osteotomy be approximately half that of the posterior side, creating a trapezoidal gap stabilized with one or two anteroposterior screws. This maneuver is designed to prevent posterior tibial slope increase. We applied this principle in our case and obtained favorable biomechanical results.

Likewise, Mabrouk et al. [19] reported a significant correlation between the anterior/posterior wedge ratio and hinge axis orientation with changes in PTS. They attributed the maintenance of patellar height and posterior slope to meticulous surgical technique, including the use of an inclined trapezoidal plate, posterior plate positioning, and fixation with the knee in extension to compress the anterior osteotomy gap. They emphasized the importance of completing the posterolateral cortical cut to properly direct the hinge anterolaterally, allowing a well-controlled anterior-to-posterior wedge opening ratio. Additional technical recommendations included: parallel guide wire placement in the sagittal plane, the most posterior plate fixation possible, complete posterolateral cortical osteotomy, and maintaining anterior wedge height at approximately half of the posterior wedge height.

Finally, some contraindications to this technique must be considered: severe bone loss in the femoral condyle or tibial plateau, presence of rheumatoid or infectious arthritis, advanced osteoarthritis in the lateral compartment, and severely limited joint mobility [8].

### 4.1 Limitations

The main limitation of this study is that it reports a single clinical case treated with an innovative surgical technique. Nevertheless, the currently available literature on this technical variant is extremely scarce and originates mostly from a single research group, reinforcing the need for independent reports that provide complementary evidence and help validate the initial published results. Another relevant limitation is the short follow-up period, which does not allow for the assessment of long-term functional and radiological outcomes. In addition, potential selection bias should be considered: the patient presented was deemed an ideal candidate for this technique, as he was a young individual (36 years old) with symptomatic varus alignment of 11.75°, advanced osteochondral damage in the medial compartment, and low patella. In this context, a valgus-producing osteotomy was required in the coronal plane without further aggravating the patellar height or altering the tibial slope. These specific characteristics may have favorably influenced the clinical outcome and limit the generalizability of the technique to patients with different profiles. However, the primary aim of this report was to demonstrate the technical feasibility and initial effectiveness of a surgical alternative specifically designed to avoid common biomechanical complications associated with conventional medial opening-wedge tibial osteotomy.

This case report has been reported in accordance with the SCARE 2025 criteria [20].

## 5 Conclusion

The biplanar “L”-shaped tibial osteotomy, by preserving the distal insertion of the patellar tendon in the proximal fragment, presents a theoretical biomechanical advantage: it allows correction of coronal malalignment without altering the posterior tibial slope or compromising patellar height in the sagittal plane. This mechanism may promote adequate joint congruence and, hypothetically, help delay the onset of degenerative changes and the progression of osteoarthritis.

In the present case, the technique enabled simultaneous correction of the deformity and treatment of the osteochondral lesion in a single-stage procedure, with a favorable short-term functional outcome. However, as this is a single case report, these findings should be interpreted with caution. Further evidence, including larger series and long-term follow-up, is required to confirm its safety, reproducibility, and effectiveness in this indication.

## Author contribution

**Dr. Eliseo Javier Firman:** Lead surgeon, manuscript writing, collection and measurement of clinical and radiological data, and patient follow-up.

**Dr. Eduardo Manuel Río:** Surgical assistance, critical revision of the manuscript, and statistical data analysis.

**Dr. Adrián Nicolás Sirio:** Surgical assistance, image analysis, clinical follow-up, significant contributions to the conception and design of the study, and intellectual review of the manuscript.

## Consent

Written informed consent was obtained from the patient for publication and any accompanying images. A copy of the written consent is available for review by the Editor-in-Chief of this journal on request.

## Ethical approval

Ethical approval is available. Although this type of publication does not typically require the Ethics section in the author disclosure form, we nonetheless submitted the study for ethical evaluation, issued by the Ethics Committee of Sanatorio Finochietto and endorsed by the Ethics Committee of the Government of the City of Buenos Aires, (Argentina), dated July 7, 2025.

## Guarantor

Dr. Eliseo Javier Firman.

## Research registration number

This study was registered retrospectively at the Research Registry (Unique Identifying Number: researchregistry11329).

## Funding

This study did not receive any specific grant, financial support, or sponsorship from public institutions, commercial entities, or non-profit organizations.

## Conflict of interest statement

The authors declare that they have no conflicts of interest.

## References

[1] Changxiao Han, Xia Li, Xiangdong Tian, Jiping Zhao, Liqun Zhou, Yetong Tan, Sheng Ma, Yuanyi Hu, Handong Chen, Ye Huang (2020). The effect of distal tibial tuberosity high tibial osteotomy on postoperative patellar height and patellofemoral joint degeneration. J. Orthop. Surg. Res..

[2] Monllau Juan Carlos, Erquicia Juan Ignacio, Ibañez Federico, Ibañez Maximiliano, Gelber Pablo Eduardo, Masferrer-Pino Angel, Pelfort Xavier (Nov 13 2017). Open-wedge valgus high tibial osteotomy technique with inverted l-shaped configuration. Arthrosc. Tech..

[3] Caton J., Deschamps G. (1982). Méthode de mesure de la hauteur de la rotule. Acta Orthop. Belg..

[4] Brazier J., Migaud H., Gougeon F., Cotton A., Fontaine C., Duquennoy A. (1996). Evaluation of methods for radiographic measurement of the tibial slope: a study of 83 healthy knees. Rev. Chir. Orthop. Reparatrice Appar. Mot..

[5] Kim Young Mo, Joo Young Bum (Dec 2012). Patellofemoral osteoarthritis. Knee Surg. Relat. Res..

[6] Takashi Habata, Kota Uematsu, Koji Hattori, Ryoji Kasanami, Yoshinori Takakura, Yoshiyuki Fujisawa (Oct 2006). High tibial osteotomy that does not cause recurrence of varus deformity for medial gonarthrosis. Knee Surg. Sports Traumatol. Arthrosc..

[7] Dwyer Tim, Ryan Martin C., Rita Kendra, Sermer Corey, Chahal Jaskarndip, Ogilvie-Harris Darrell (2017). Reliability and validity of the arthroscopic International Cartilage Repair Society classification system: correlation with histological assessment of depth. Arthroscopy.

[8] Erquicia Juan, Gelber Pablo E., Perelli Simone, Ibañez Federico, Ibañez Maximiliano, Pelfort Xavier, Monllau Juan Carlos (2019). Biplane opening wedge high tibial osteotomy with a distal tuberosity osteotomy: radiological and clinical analysis with minimum follow-up of 2 years. J. Exp. Orthop..

[9] Resch T., Szymski D., Hartz F., Zehnder P., Römmermann G., Angele P. (Jul 2024). Open-wedge high tibial osteotomy with and without bone void filler: allograft leads to faster bone union and weight bearing with comparable return to work and sports rates. Knee Surg. Sports Traumatol. Arthrosc..

[10] Garabano G., Perez Alamino L., Rodriguez J., Alonso M., Pesciallo C.A. (Jul 2024). Maintenance of joint reduction and allograft incorporation in treating tibial plateau fractures: retrospective case series using cortico-cancellous, non-irradiated bone allograft. J. Clin. Orthop. Trauma.

[11] Mao Y., Yao L., Li J., Li J., Xiong Y. (Feb 2024). No superior bone union outcomes with allografts compared to no grafts and autografts following medial opening wedge high tibial osteotomy: a retrospective cohort study. Orthop. Surg..

[12] Bodenbeck E.M., Böpple J.C., Doll J., Bürkle F., Schmidmaier G., Fischer C. (Jan 2024). Earlier consolidation and improved knee function of medial open wedge high tibial osteotomy with autologous bone graft. Eur. J. Orthop. Surg. Traumatol..

[13] Yang J.C., Lobenhoffer P., Chang C.M., Chen C.F., Lin H.C., Ma H.H., Lee P.Y., Lee O.K. (Dec 30 2020). A supplemental screw enhances the biomechanical stability in medial open-wedge high tibial osteotomy. PLoS One.

[14] Chang C.W., Chen Y.N., Li C.T., Chung C.R., Tseng C.C., Chang C.H., Peng Y.T. (Feb 2019). Biomechanical investigation of tibial tubercle osteotomy fixed with various screw configurations. Injury.

[15] Umut Öktem, Suha Dedeoğulları Emin, İzzet Bingöl, Saygin Kamacı, İbrahim Bozkurt, Ali Öçgüder Durmus (Feb 13 2025). Effect of posteriorly inclined sagittal osteotomy on posterior tibial slope in biplanar medial opening wedge high tibial osteotomy: a case series study. BMC Musculoskelet. Disord..

[16] Keliang Wu, Jianchun Zeng, Linjing Han, Wenjun Feng, Xiaosheng Lin, Yirong Zeng (2021). Effect of the amount of correction on posterior tibial slope and patellar height in open-wedge high tibial osteotomy. J. Orthop. Surg..

[17] MacLeod Alisdair R., Roberts Samuel A., Gill Harinderjit S., Mandalia Vipul I. (Dec 2023). A simple formula to control posterior tibial slope during proximal tibial osteotomies. Clin. Biomech. (Bristol).

[18] Chung Jai Hyun, Choi Chong Hyuk, Kim Sung Hwan, Kim Sung Jae, Suk Yong June, Jung Min (2022). Effect of the sagittal osteotomy inclination angle on the posterior tibial slope change in high tibial osteotomy: three-dimensional simulation study. Sci. Rep..

[19] Ahmed Mabrouk, Jae-Sung An, Reina Fernandes Levi, Kristian Kley, Christophe Jacquet, Matthieu Ollivier (Dec 11 2023). Maintaining posterior tibial slope and patellar height during medial opening wedge high tibial osteotomy. Orthop. J. Sports Med..

[20] Kerwan A., Al-Jabir A., Mathew G., Sohrabi C., Rashid R., Franchi T., Nicola M., Agha M., Agha R.A. (2025). Revised Surgical CAse REport (SCARE) guideline: an update for the age of Artificial Intelligence. Prem. J. Sci..

